# Indigestion and Its Treatment

**Published:** 1886-03

**Authors:** 


### "Indigestion," and its Treatment.

A writer in the *London Medical Times* (July 4, 1885), says: There is no complaint of which the diagnosis and the treatment are more often vitiated by gratuitous theorizing, and consequently meddlesome drugging, than chronic indigestion. Moreover, this same love of theory and "treatment"—a curious source of ready satisfaction to many medical minds—leads to a great exaggeration of the number of cases which are dubbed dyspeptic. Satisfied with a supposition of some derangement of the secretions or movements of the stomach which can not be proved or observed, but is all the more elaborately and dogmatically enunciated, too many of us are apt to search no further than this organ for the ultimate cause of the complaint which we have to treat. And yet it may be boldly stated that an immense majority of the patients who come to doctors for relief from pain, or discomfort of some kind, be it "fullness," or flatulence, or flushing, after taking their meals, have little or nothing the matter primarily with their stomachs, and do not require, but should rather avoid, any medicine directed thereto, or indeed any drug at all. This proposition will be found to be true just in proportion to the *apparent* freedom of these patients from any ailment except that of the stomach. In considering this question, cases of organic disease, as cancer, or ulcer, or gastric catarrh from obvious irritatives, such as alcohol, or patently immoderate living, are left out of sight; for they are evidenced by other symptoms beyond those to which we refer, and, generally revealing themselves to the careful medical enquirer, are not ticketed as dyspepsia. Equally of course are ignored the common derangements of the stomach which would more properly be termed acute, occurring in the course of fevers and inflammations, or as the immediate results of acts of gluttony or drunkenness. The *ordinary* dyspeptic patient is one who suffers at certain times after taking food; and even if never quite free from his complaint, which is not often the case, is at all events much worse at such times than at others. Many, indeed, both claim and appear to be quite well except at the fatal after-dinner time.

To come to a correct conclusion as to the diagnosis and treatment of these cases, a comprehensive knowledge is brought into requisition, and the more such knowledge is practically applied, the smaller will be the number of patients who will be vaguely called dyspeptics. We shall then eliminate the gouty, and the sufferers from the less obvious forms of renal disease, aud what is often lost sight of, the victims of unsuspected cardiac mischief. It is not altogether rare to find patients persistently treated, and treated in vain, for "dyspepsia" by so-called stomachic remedies, be they acids, or alkalies, or bismuth, or the many artificial and almost useless so-called physiological "digestives" the names of which end in *-pepsin*, whose symptoms, though incidentally referrable to gastric congestion, are really due to disease of the heart, and especially of the mitral valve. Again, the sufferers from chronic pulmonary affections, and notably from bronchitis, furnish a considerable contingent of cases which for the patients' sakes should not be put down as "indigestion." The host of neurotics, whose prominent complaint is dyspepsia, will certainly be more successfully treated by general hygienic or "tonic" methods than by "stomachic" medicines, or indeed, very often, than by giving much attention to even dietetic rules; and a change of habits or social surroundings will frequently succeed where all the resources of the cook and the druggist have been taxed in vain by the "therapeutical"' physician. Psychical dyspeptics are misunderstood and maltreated by thousands.

It would seem perhaps scarcely necessary to insist upon the all-important part played in the production of some of the most troublesome cases of indigestion by the deficiency or absence of teeth. But this cause is surprisingly often overlooked by the most ardent treaters of disease, and cases might be quoted in numbers where many doctors, including those the most eminent "for the stomach," have vainly drugged for years a healthy organ, whose owner has been at last relieved of his intolerable sufferings by the art of the dental mechanician.

Most prominent of all perhaps among the dyspeptics who apply for relief are those whose sufferings, sometimes of great severity, are due only to neglect of proper intervals between their feeding-times. Not quality or quantity of food is here at fault at all. These are they who take good breakfasts, because their digestive organs have had their physiological and therefore unstarved rest; who do not suffer much or at all after luncheon (if they take any); but from an over-late dinner, when their possibly idiosyncratic stomachs have been long empty, undergo tortures which only an unnatural appetite can drive them to endure. Drugs are not good for such as these.

From this small and imperfect selection of common-place sketches from life it will be seen that "dyspepsia" ought not to be so great a bugbear to the doctor as it often appears to be. The despair of the therapeutist should decrease with his dwindling drug-list. Useful as in very many cases of organic disease of the stomach various medicines are, and indeed indispensable as perhaps the only means of relieving the patient, they are in such cases only palliative; and the same, in a modified sense, may be said of most of the remedies in the forms of acids and alkalies which are given at certain times and with certain rules, with the often plausible notion of affecting the secretions of the stomach. A dose of such medicine may relieve the pain or discomfort which follows a meal, or may even, though more doubtfully, prevent such pain in rare cases by promoting digestion; but the effect is only temporary; the cause is not touched, and the theory which prompts their administration is elaborated more in the study than at the bedside. The stomach rarely suffers alone; never, perhaps, in indigestion which is *curable.* In curable dyspepsia drugs reach their lowest, in incurable disease of the stomach their highest, point of utility. Pepsin may relieve discomfort for the moment; but it seldom does, and at best it is a sorry therapeutic crutch. Opium, and even bismuth, are often a god-send to a cancerous stomach. And with respect to the purely dietetic treatment of dyspepsia, there are two sides of the question. Doubtless the dyspeptic should not disobey the accepted dicta of physiology and of general experience with regard to his manner of living; and broad rules should in most cases be enforced. But in proportion as a man is dyspeptic, so he often finds out best for himself what to take and what to avoid; and as long as his meals are moderate in quantity and regular in time, he will often be better with a comparatively unrestricted diet-table. The most "dietetic" of doctors rarely practice themselves what they preach to the healthy, and the least dyspeptic of dispeptics are verily not those who are always taking thought for their daily meat and drink.

